# Nanotechnology: A Valuable Strategy to Improve Bacteriocin Formulations

**DOI:** 10.3389/fmicb.2016.01385

**Published:** 2016-09-16

**Authors:** Hazem A. Fahim, Ahmed S. Khairalla, Ahmed O. El-Gendy

**Affiliations:** ^1^Department of Biotechnology and Life Sciences, Faculty of Post Graduate Studies for Advanced Sciences, Beni-Suef UniversityBeni-Suef, Egypt; ^2^Department of Microbiology and Immunology, Faculty of Pharmacy, Beni-Suef UniversityBeni-Suef, Egypt

**Keywords:** bacteriocin, antimicrobial peptides, biopreservative, nanotechnology, nanoformulated bacteriocins, drug delivery systems

## Abstract

Bacteriocins are proteinaceous antibacterial compounds, produced by diverse bacteria, which have been successfully used as: (i) food biopreservative; (ii) anti-biofilm agents; and (iii) additives or alternatives to the currently existing antibiotics, to minimize the risk of emergence of resistant strains. However, there are several limitations that challenge the use of bacteriocins as biopreservatives/antibacterial agents. One of the most promising avenues to overcome these limitations is the use of nanoformulations. This review highlights the practical difficulties with using bacteriocins to control pathogenic microorganisms, and provides an overview on the role of nanotechnology in improving the antimicrobial activity and the physicochemical properties of these peptides.

## Introduction

Bacteriocins are a group of polypeptides that are produced by a variety of Gram-negative and Gram-positive bacteria, and exhibit bactericidal or bacteriostatic activity, usually against species closely related to the producing strain (Tagg et al., 1976; Castellano et al., 2012; El-Gendy et al., 2013). While they may be categorized as antibiotics, bacteriocins differ in that they are: (i) proteinaceous, ribosomally synthesized, molecules produced during the primary phase of growth; (ii) known to exhibit a relatively narrow spectrum of antibacterial activity; (iii) inactivated by digestive enzymes, which makes them non-toxic to human cells if used as biopreservative; and (iv) unique in their mechanism of action (Tagg et al., 1976; Zacharof and Lovitt, 2012; Balciunas et al., 2013; Perez et al., 2014). Regarding the structure, classification, mode of action, and genetic characterization of bacteriocins, these aspects have been discussed in a number of reviews (Klaenhammer, 1993; Héchard and Sahl, 2002; Nes et al., 2007; Hoover and Steenson, 2014) and will not be covered here.

Over the last decade, bacteriocins have gained considerable attention due to their potential applications in the food industry as natural biopreservatives, and more recently in the health industry as antimicrobial agents (Zacharof and Lovitt, 2012; El-Gendy et al., 2013). Regarding the first field of application, bacteriocins can be either added directly as purified (or partially purified) agents to food or produced through cultivation of the bacteriocin-producer strain in the food substrate (Deegan et al., 2006). Among the advantages gained by this approach are: chemical-free preservation, shelf-life extension, and inhibition of food-borne pathogenic bacteria during the farming and food-processing stages (Deegan et al., 2006). Moving to the second field of application, the expanding clinical applications of bacteriocins may help us to fill some gaps in the biomedical sector. For example, some bacteriocins have displayed activity against Gram-positive pathogens of human and animal origin, including methicillin-resistant *Staphylococcus aureus* (MRSA), and vancomycin-resistant *Enterococcus faecalis* strains (Kruszewska et al., 2004; Millette et al., 2008). This antibacterial activity makes bacteriocins a promising substitute or a synergistic component to the currently used antibiotics to overcome the emergency of bacterial resistance.

However, in spite of these promising advantages, nisin is the only bacteriocin generally recognized as safe by the Food and Drug Administration and is currently used as a food preservative in several countries (Delves-Brougthon, 1990; Montville and Chen, 1998). This limitation in bacteriocins availability in the market as preservatives and antimicrobials can be attributed to multiple factors, including: (i) the high cost of their commercial production (Bradshaw, 2003); (ii) the loss of their activity by proteolytic enzymes (Bradshaw, 2003); (iii) their unfavorable interactions with other food constituents, which decreases the availability and necessitates a huge amount of the peptide to be added (Jung et al., 1992; Schillinger et al., 1996); (iv) the alterations of the chemical and physical properties of these compounds during the various food-processing stages (Davidson et al., 2005); (v) the low yield of these compounds due to ineffective recovery by traditional purification methods (Carolissen-Mackay et al., 1997); and (vi) the narrow spectrum of activity observed for most of the tested bacteriocins against pathogenic bacteria (Riley and Wertz, 2002). In the last years, several studies on bacteriocins have demonstrated that the optimization of their production conditions, their purification methods, their combinations with other antimicrobial agents, and the hurdle technology approach, could all represent solutions to some of the previously mentioned problems (Kalchayanand et al., 1994; Li et al., 2001; Wolska et al., 2012; Saraniya and Jeevaratnam, 2014). While scientists are searching for efficient strategies to overcome the limitations of bacteriocins, the use of nanotechnology is a potential approach to maximize the use of these peptides (Allémann et al., 1998; Salmaso et al., 2004). Therefore, this article aims to elucidate the current applications of nanotechnology in improving the properties and the antimicrobial activity (AMA) of bacteriocins.

## Advantages of nanoformulated bacteriocins

**Figure 1:**
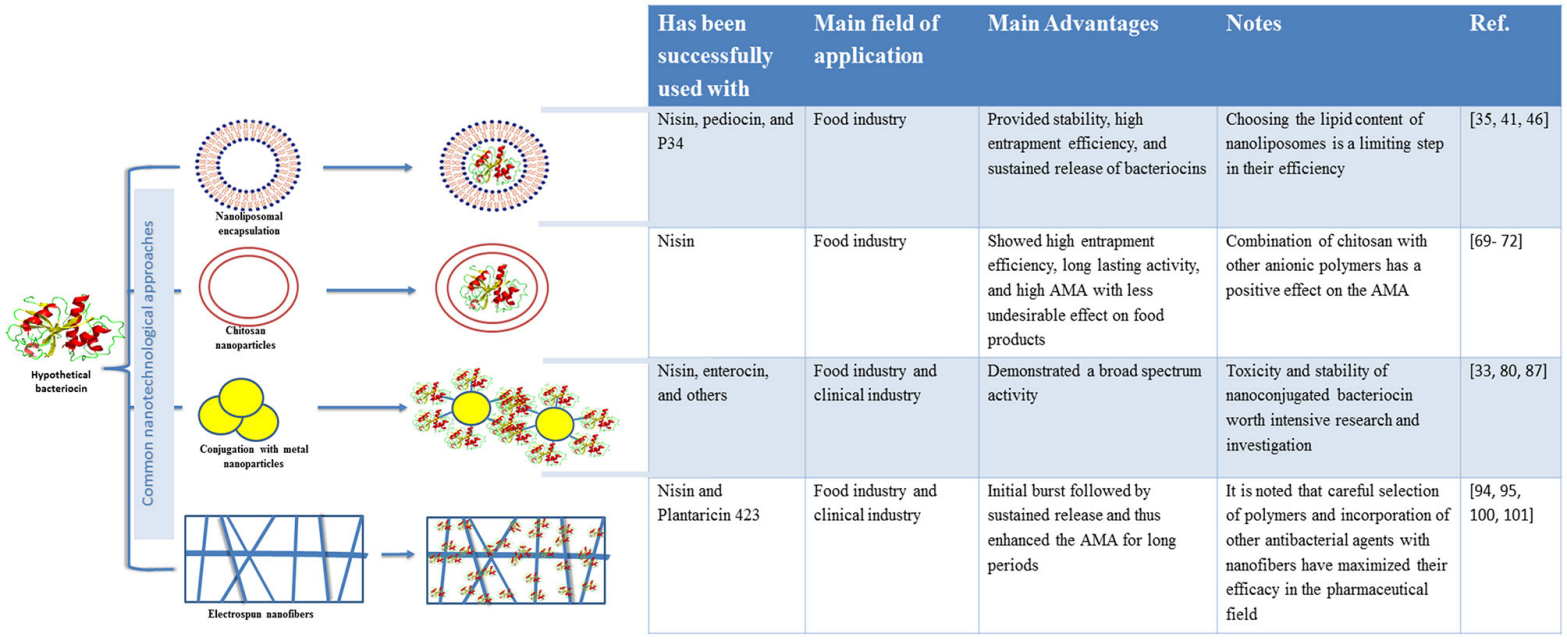
**Schematic representation showing some of the nanotechnological approaches used in bacteriocin formulations with their associated characters**. 35, da Silva Malheiros et al., 2012a; 41, da Silva Malheiros et al., 2012b; 46, de Mello et al., 2013; 69, Zohri et al., 2010; 70, Zohri et al., 2013;71, Bernela et al., 2014; 72, Chopra et al., 2014; 33, Mossallam et al., 2014; 80, Thirumurugan et al., 2013; 87, Sharma et al., 2012; 94, Heunis et al., 2013; 95, Heunis et al., 2011; 100, Ahire and Dicks, 2015; 101, Ahire et al., 2015.

According to the U.K. House of Lords Science and Technology Committee, nanotechnology is the manipulation of functional materials and structures into the nanoscale size (with diameters ranging from 1 to <1000 nm; Klaessig et al., 2011). This is a quite novel technology that has several applications in various fields of science due to the unique features of the synthesized nanoparticles (Chou et al., 2011). The integration of nanotechnology and biotechnology opens the door to unlimited opportunities and future perspectives to solve the problems belonging to a range of biological products. Through this integration, effective delivery, targeting, protection from degradation, in addition to improving drug potency and physicochemical properties can all be achieved (Farokhzad and Langer, 2009). Bacteriocins are one of the many examples that can benefit from such combination. For instance, nano-encapsulation of bacteriocins intended for use as biopreservatives could protect them from degradation by proteolytic enzymes, in addition to rescuing them from undesirable interactions with other food components, and hence, increasing their stability for longer periods (Brandelli, 2012). Furthermore, some recent studies have shown that encapsulation of bacteriocins in nanoparticles has enhanced the activity of these peptides against food-spoiling microorganisms and multidrug-resistant bacteria (Arthur et al., 2014; Mossallam et al., 2014). In addition, the use of nanotechnology-based materials and/or methods has, in most cases, shown a positive impact on bacteriocin yield, thus facilitating their commercial production (Zacharof et al., 2013). Table 1 illustrates several examples of bacteriocins that have been formulated using nanotechnological approaches, while Figure 1 summarizes the major benefits form such formulations. However, it must be stated that some of the nanoformulated bacteriocins have nearly the same activity (or even lower) compared with the free ones (da Silva Malheiros et al., 2012a; Malheiros Pd et al., 2012), which will be explained below in more details.

**Table 1:** **Examples of bacteriocins that have been formulated using nanotechnological approaches**.

**Bacteriocin**	**Nanotechnological approach**	**Fabrication method**	**Characters of the resulting nanoformulation**		**Tested microorganism(s)**	**Effect(s) of nanoformulation**	**References**
			**Particle size**	**Entrapment efficiency (%)**			
BLS P40 produced by *Bacillus licheniformis*	Phosphatidylcholine nanovesicles	Reverse phase evaporation method	570 nm	NR	*Listeria monocytogenes*	Maintained the AMA for a longer period	Teixeira et al., 2008
BLS P34	Phosphatidylcholine nanovesicles	Thin-film hydration method	160 nm	100%	*L. monocytogenes*	Both the free and the encapsulated bacteriocins had nearly the same AMA	da Silva Malheiros et al., 2012a
Bacteriocin produced by *Lactobacillus plantarum* ATM11 and nisin	Gold nanoparticles	NR	NR	NR	*Bacillus cereus, Escherichia coli, S. aureus*, and *Micrococcus luteus*	Enhanced the AMA against some food spoiling microorganisms	Thirumurugan et al., 2013
Enterocin	Silver nanoparticles	NR	325 nm	NR	A group of Gram-positive and Gram-negative bacteria	Demonstrated broad-spectrum inhibition against a group of food pathogens without any detectable toxicity to red blood cells (RBCs)	Sharma et al., 2012
Bacteriocin produced by *Lactobacillus acidophilus* CH1	Gold nanoparticles	NR	20.15 nm	NR	*Enterocytozoon bieneusi* spores	Increased the anti-microsporidial effect without significant cell toxicity	Mossallam et al., 2014
Nisin	Phosphatidylcholine Nanoliposomes	NR	144, 167, and 223 nm depending on the type of nanoliposomes	54–63%	NR	Nisin entrapped efficiently in nanoliposomes	Were et al., 2003
Nisin	Nanoliposomes	NR	Different particle size due to differences in preparation methods	70–90%	NR	Provided stability to a wide range of temperature conditions	Taylor et al., 2007
Nisin	Phosphatidylcholine nanoliposomes	Reversed-phase and hydration film methods	190, 181 and 148 nm depending on the preparation method	94.12% with film hydration method	*L. monocytogenes*	The free nisin was more potent and exhibited more sustained release compared to the encapsulated one	da Silva Malheiros et al., 2010a
Nisin	Phosphatidylcholine nanoliposomes	Thin-film hydration method	140 nm	100%	*L. monocytogenes*	The free nisin was more potent than the encapsulated one	Malheiros Pd et al., 2012
Nisin A	Phosphatidylecholine nanoliposomes	Thin-film hydration method	140 nm	94%	*L. monocytogenes*	Both the free and the encapsulated bacteriocins had nearly the same AMA at low temperature	da Silva Malheiros et al., 2010b
Nisin and BLS P34	Phosphatidylecholine nanoliposomes	Thin-film hydration method	218 nm for nisin, and 158 nm for BLS P34	88.9% for nisin and 100% for BLS P34	*L. monocytogenes*	Displayed higher AMA	da Silva Malheiros et al., 2012b
Nisin Z	Nanoliposomes	NR	Different particle size (190–295 nm) depending on the type of nanoliposome	12–54%	*Bacillus subtilis* and *Pseudomonas aeruginosa*	Exhibited stability for several months	Colas et al., 2007
Nisin	Solid lipid nanoparticles (SLN)	High pressure homogenization	159–175 nm depending on the concentration of nisin	69.2–73.6%	*L. monocytogenes* and *L. plantarum*	Extended the AMA for a longer duration	Prombutara et al., 2012
Nisin	Chitosan / alginate nanoparticles	NR	50–205 nm	90–95%	*S. aureus*	Maximized and prolonged the AMA with minimum concentration of nisin	Zohri et al., 2010
Nisin	Chitosan / alginate nanoparticles	NR	205 nm	NR	*L. monocytogenes* ATTC 25923 and *S. aureus* ATTC 19117	Enhanced the AMA to a higher extent with less damaging effect on the tested food samples	Zohri et al., 2013
Nisin	Chitosan / carageenan nanocapsules	Ionic complexation method	397.6–1106 nm	53–93.32%	*Micrococcus luteus* MTCC 1809, *P. aeruginosa* MTCC 424, *Salmonella enterica* MTCC 1253, and *Enterobactor aerogenes* MTCC 2823	Demonstrated long-lasting AMA	Chopra et al., 2014
Nisin	Tripolymeric nanoformulation prepared from chitosan, sodium alginate and pluronic F68	Ionotropic pre-gelation method followed by polycationic crosslinking	130–178 nm	41.45–88.36%	*M. luteus* MTCC 1809, *P. aeruginosa* MTCC 424, *S. enterica* MTCC 1253 and *Enterobactor aerogenes* MTCC 2823	Encapsulated nisin released in a sustained manner and displayed AMA for a longer period	Bernela et al., 2014
Nisin	Carbohydrate nanoparticles	NR	NR	NR	*L. monocytogenes*	Extended the AMA for a longer period of time	Bi et al., 2011a
Nisin	Carbohydrate nanoparticles	Adsorption of nisin to emulsion of nanoparticles	336 and 50.2 nm depending on the type of phytoglycogen	NR	*L. monocytogenes*	Retained the efficacy for a longer period of time	Bi et al., 2011b
Nisin	Nanofibers	Electrospining process	330 ± 79 nm	NR	*S. aureus*	Prolonged the antimicrobial activity against skin infection and accelerated the wound healing	Heunis et al., 2013
Nisin	Nanofibers	Electrospinning process	200–250 nm	NR	A strain of MRSA	Increased the AMA in presence of 2,3-dihydroxybenzoic acid	Ahire and Dicks, 2015
Nisin	Nanofibers with Silver nanoparticles	Electrospinning process	288 ± 63 nm	NR	*S. aureus, P. aeruginosa, Klebsiella pneumonia, E. coli, and S. typhimurium*.	Provided a broad spectrum AMA	Ahire et al., 2015
Nisin	Poly-L-lactide (PLA) nanoparticles	Semi-continuous compressed CO_2_ anti-solvent precipitation	200–400 nm depending on the concentration of nisin	About 95%	*Lactobacillus delbrueckeii*	Extended the AMA for a longer duration	Salmaso et al., 2004
Pediocin	Phosphatidylecholine nanoliposomes	Thin-film hydration method with bath-type sonicator	190 nm	80%	*L. monocytogenes*	Encapsulated pediocin maintained the AMA for a longer period of time, but the free one was more potent	de Mello et al., 2013
Plantaricin 423	Nanofibers electrospining	Electrospining process	288 nm	NR	*Lactobacillus sakei*, and *Enterococcus faecium*	Decreased the AMA	Heunis et al., 2010
Plantaricin 423 and bacteriocin ST4SA	Nanofibers electrospining	Electrospining process	200–450 nm	NR	*E. faecium* and *L. monocytogenes*	Displayed higher AMA for a longer period	Heunis et al., 2011
The antimicrobial peptide P34	Nanoliposomes	Thin-film hydration method	150 nm	100%	*L. monocytogenes*	Both the free and the encapsulated P34 showed nearly the same AMA	da Silva Malheiros et al., 2011

BLS: bacteriocin-like substance; NR: not reported by authors.

## Nanotechnological approaches used in bacteriocin formulations

### Encapsulation in lipid-based nanoparticles

#### Nanoliposomes

Liposomes are spherical structures composed of single or multiple phospholipid bilayer membranes enclosing an aqueous medium with a size ranging from nanometer to micrometer (Bangham et al., 1965; Vemuri and Rhodes, 1995). Besides being non-toxic, liposomes are biodegradable agents suitable for encapsulating both hydrophilic and hydrophobic substances (Gundermann and Schumacher, 1990; Kulkarni et al., 1995). Especially when prepared at the nano size, nanoliposomes represent a promising vehicle for the encapsulation and delivery of several drugs and bioactive compounds, including bacteriocins (Banerjee, 2001; Mugabe et al., 2005; da Silva Malheiros et al., 2012b). For example, phosphatidylcholine-based nanoliposomes have been shown to exhibit high entrapment efficiency (almost 100%) for the bacteriocin-like substance (BLS) P34, without having a negative effect on its antimicrobial activity (da Silva Malheiros et al., 2012a). In another study, nanoliposomes prepared from different lipid/phospholipid compositions and ratios were tested for their: (i) capacity to encapsulate nisin Z; (ii) stability; and (iii) bacterial targeting (Colas et al., 2007). The highest entrapment efficiency for nisin Z (54.2%) has been recorded for nanoliposomes composed of dipalmitoylphosphatidylcholine/dicetylphosphate/cholesterol (DPPC:DCP:CHOL) with 7:2:1 molar ratio (Colas et al., 2007). Additionally, nanoliposomes prepared from DPPC:DCP:CHOL (at 7:2:1 ratio) or from dipalmitoylphosphatidylcholine/stearylamine/cholesterol (at 7:2:1 ratio) have been shown to possess the highest stability, which extended for 14 months at 4°C in case of the former, and 12 months at 25°C in case of the latter (Colas et al., 2007). The results have also shown the ability of nanoliposomes prepared from DPPC:DCP:CHOL to target the outer membranes of *Bacillus subtilis* (Colas et al., 2007). A study similar to the one just described was conducted by Taylor et al. (2007), in which nanoliposomes prepared from distearoylphosphatidylcholine (PC) and distearoylphosphatidylglycerol (PG) were used to encapsulate nisin. Their results have shown the ability of liposomes consisting of PC, PC/PG (at 8:2), and PC/PG (at 6:4 ratio) to retain about 70–90% of the incorporated nisin with high stability, despite exposure to elevated temperatures (25°–75°C) and acidic or alkaline pH [46]. These findings suggest that the stability of bacteriocins against unfavorable environmental conditions can be improved by nanoliposomal formulations, which still remains to be proved by assessing their AMA (Taylor et al., 2007). A similar study was reported by de Mello et al. (2013), in which pediocin AcH had been successfully loaded with high (80%) entrapment efficiency, stability, and antimicrobial activity, for at least 13 days, when incorporated into phosphatidylcholine nanovesicles (de Mello et al., 2013). However, it must be stated that while the liposome-encapsulated pediocin has been shown to maintain its AMA for a longer period, this activity was lower than that of the free pediocin, which requires further investigations to overcome this issue (de Mello et al., 2013). These collective studies indicate that nanoliposomal formulations of bacteriocins may have distinct capacities to withstand environmental and chemical stresses typically encountered during the different food-processing stages.

Protection from degradation and enhancement of stability are not the only advantages of liposome-encapsulated bacteriocins, as some of these formulations have exhibited better AMA, in terms of either spectrum or duration, which will be discussed in more details in the following paragraph. For instance, phosphatidylcholine nanovesicles containing BLS produced from *Bacillus licheniformis* P40 have been shown to completely inhibit *L. monocytogenes* within the first 12 min of incubation (Teixeira et al., 2008). In terms of duration of activity, while the encapsulated BLS has been shown to maintain its initial AMA over 30 days of incubation, it has taken only 14 days for the free one to lose 90% of its activity (Teixeira et al., 2008). In terms of safety, the encapsulated BLS has been shown to lack any hemolytic activity on human erythrocytes, suggesting its safety as food biopreservative (Teixeira et al., 2008). In another study, BLS P34 and nisin were encapsulated individually in nanoliposomal formulations prepared either from phosphatidylcholine alone or phosphatidylcholine/cholesterol (at 7:3 ratio; da Silva Malheiros et al., 2012b). While all the tested formulations have inhibited *L. monocytogenes* growth, the phosphatidylcholine-based preparations that have been stored for 10 days demonstrated the highest AMA against this bacterium in Minas frescal cheese samples (da Silva Malheiros et al., 2012b). Therefore, phosphatidylcholine nanovesicles seem to promote the slow release of the incorporated bacteriocins, which explains the storage duration required to exert their efficacy against target microorganisms (da Silva Malheiros et al., 2012b).

However, in spite of the above encouraging examples, encapsulated bacteriocins with activity similar to, or less than, the free ones have been reported in few other studies (da Silva Malheiros et al., 2012a; Malheiros Pd et al., 2012). For example, when nisin has been encapsulated in phosphatidylcholine nanoliposomes, the encapsulated and the free nisin had both displayed nearly equal antilisterial activities (da Silva Malheiros et al., 2010a). Additionally, while the free nisin has been shown to maintain its AMA over 24 days of incubation, the activity of the encapsulated one has declined to 25% of its initial levels after only 10 days of storage (da Silva Malheiros et al., 2010a). Another example is the study of Malheiros Pd et al. (2012), in which nisin encapsulated in phosphatidylcholine nanoliposomes has only displayed a bacteriostatic effect against *L. monocytogenes* in Minas frescal cheese, whereas the free nisin has exhibited a bactericidal effect under the same conditions (da Silva Malheiros et al., 2010a). This negative impact on bacteriocin AMA may be attributed to: (i) selection of unsuitable phospholipid-bacteriocin combinations; (ii) undesirable interactions between the bacteriocin and the phospholipid forming the liposome; (iii) stresses applied during the encapsulation process; and/or (iv) low-purity level of the starting materials, including the antimicrobial peptide itself (Were et al., 2003; da Silva Malheiros et al., 2010a,b). Therefore, further studies are required to optimize the formulation of bacteriocin-loaded liposomes, in order to avoid the negative impacts mentioned above.

#### Solid lipid nanoparticles (SLN)

Another example of lipid-based formulation systems is SLN, in which each nanoparticle is composed of a triglyceride core with a phospholipid coat of high-melting point, which is responsible for keeping them in a solid state, both at room and human body temperatures (Puri et al., 2009). In addition to the multiple advantages of liposomes, the solid core possessed by SLN makes them powerful tools for large-scale production and slow-release drug formulations (Feng and Mumper, 2013). In a recent study, the release of nisin incorporated into SLN carrier has continued for about 25 days, depending on the pH and the salt concentration of the buffer solution (Prombutara et al., 2012). Additionally, nisin-loaded SLN have demonstrated activity against *L. monocytogenes* DMST 2871 for up to 20 days and activity against *L. plantarum* TISTR 850 for up to 15 days, while the activity of free nisin lasted only for 3 days against the former organism and for 1 day against the latter (Prombutara et al., 2012). This indicates that SLN have the ability to protect bacteriocins from degradation, and hence extend their antibacterial activity for a longer period of time. However, the use of SLN as a delivery system for bacteriocins is still in the early exploratory phases of research. Furthermore, there are a number of challenges that need to be overcome to fully establish the SLN as a delivery system; these challenges include the possible expulsion of the incorporated drug/drug-like agents from the lipid matrix and the low drug-loading capacity (Jenning et al., 2000; Souto et al., 2006).

### The use of carbohydrate-based nanoparticles

#### Chitosan/alginate nanoparticles

Carbohydrates are naturally occurring organic substances that serve both structural and storage functions (Ghazarian et al., 2011). They are biodegradable, biocompatible substances, with highly stable properties, and thus have attracted much attention for their applications in the food, biomedical and environmental fields (Chen and Soucie, 1985; Jizomoto et al., 1993; Richardson et al., 1999; Melamu and Von Blottnitz, 2009). Chitosan, a natural biopolymer produced by the deacetylation of chitin, is one of the most commonly used polysaccharides for fabrication of nanoparticles (Nitta and Numata, 2013). In addition to being non-toxic, biodegradable, and biocompatible, chitosan is characterized by its antibacterial activity, together with its ability to deliver drug molecules and biological compounds to their target destination (Richardson et al., 1999; Jia et al., 2001). For example, chitosan nanoparticles have demonstrated high efficiency for the delivery of diverse compounds, such as insulin, genes, vaccines, and other molecules (Vila et al., 2004; Lavertu et al., 2006; Li et al., 2009; Zhang et al., 2010). The combination of chitosan and alginate has been shown to improve the characters of both polymeric components and to provide better delivery than that obtained by using each biopolymer separately (Murata et al., 1993; Sezer and Akbuga, 1999). Such combination has been successfully used to encapsulate nisin, with 95% entrapment efficiency (Zohri et al., 2010). The encapsulated nisin has been found to be released in high concentrations within the first 4 h, followed by a steadily sustained release for more than 5 h (Zohri et al., 2010). Regarding its biological activity, the nisin-loaded chitosan/alginate has exhibited a much higher level of AMA (about 2-folds higher) than that of the free nisin, when tested against *S. aureus* ATCC 19117 (Zohri et al., 2010). Also, the minimum inhibitory concentration (MIC) of the nisin-loaded nanoparticles has been shown to be four times less than that of the free nisin (0.5 and 2 mg/ml, respectively; Zohri et al., 2010). Additionally, the nisin-loaded nanoparticles have shown significant growth-suppressing effects on *S. aureus* in both raw and pasteurized milk samples, which remained for at least 24 and 48 h, respectively, compared to 14 and 24 h in case of the free nisin (Zohri et al., 2010). These promising results have been emphasized in another study performed by Zohri et al. (2013). In their study, the nisin-loaded chitosan-alginate nanoparticles have shown a higher level of AMA against *L. monocytogenes* and *S. aureus* compared with the free nisin (Zohri et al., 2013). Furthermore, this nano-polymer hybrid did not affect the physicochemical characters of the tested food material (Zohri et al., 2013). Similar studies have demonstrated the efficiency of chitosan-based delivery systems for bacteriocins (Bernela et al., 2014; Chopra et al., 2014). Among the advantages provided by using this type of nanodelivery systems are: the potent, long-lasting AMA, the sustained-release characteristics of the system, and the maintenance of original food quality (Bernela et al., 2014; Chopra et al., 2014). Therefore, it can be concluded that harnessing of these biocompatible nanoparticles in the food industry is a promising strategy for delivery of natural food preservatives in high efficiency with fewer undesirable effects.

#### Phytoglycogen nanoparticles

Phytoglycogen is a polysaccharide material found in plants, which is commonly used for preparing novel functional nanoconstructs (Chen et al., 2015). In addition to chitosan that has been discussed earlier, phytoglycogen and its derivatives represent another class of carbohydrate-based nanoparticles that have been successfully used as carriers for nisin (Bi et al., 2011a). When the capabilities of different phytoglycogen derivatives have been examined as carriers of nisin, all the derivatives have demonstrated a long-lasting AMA against *L. monocytogenes*, but the longest activity has been associated with octenyl succinate and β-amylolysis substitutions (Bi et al., 2011a). Both phytoglycogen derivatives have retained the activity of nisin against common food pathogenic bacteria for 21 days, in comparison with 7 days in case of the free nisin (Bi et al., 2011a). In a similar study, phytoglycogen octenyl succinate has been effectively used to form an oil-in-water emulsion for delivering nisin against *L. monocytogenes* (Bi et al., 2011b). The antibacterial activity of this nanoparticle-stabilized emulsion has been higher than that of the free nisin during 50 days of storage (Bi et al., 2011b). Overall, these results encourage researchers to exploit nanomaterials as carriers for bacteriocins, which may be especially beneficial to the food industry, to ensure the safety of food both at the packaging stage and after opening the package.

### Conjugation with nanosized metals

#### Conjugation with gold nanoparticles

Metal nanoparticles as gold, silver, copper, zinc have shown potent AMA against pathogenic bacteria (Yoon et al., 2007; Kuo et al., 2009; Raghupathi et al., 2011). Generally, this is due to the large surface area of these positively charged nanoparticles, which facilitates their binding to the negatively charged bacterial membrane (Seil and Webster, 2012). The targeted bacteria are then killed by the oxidative stress induced by the generated reactive oxygen species, together with the toxicity of the accumulated free metal ions (Seil and Webster, 2012). This proposed mechanism of action sets metal nanoparticles as a promising approach to solve the problem of antimicrobial resistance. Therefore, combinations of bacteriocins and nanosized metals are expected to have a synergistic effect on antibacterial properties (Thirumurugan et al., 2013). In a recent study, conjugates containing gold nanoparticles with either nisin or a bacteriocin produced by *L. plantarum* ATM11 have both displayed significant AMA compared with the free bacteriocins, especially against *M. luteus, B. cereus, E. coli*, and *S. aureus* (Thirumurugan et al., 2013). This demonstrates the efficiency of such combinations in extending the shelf-life of food products by inhibiting a number of common food-spoilage microorganisms. In a similar study, incorporation of a bacteriocin produced by *L. acidophilus* CH1 with gold nanoparticles has resulted in a formulation with potent activity against intestinal microsporidiosis in immunocompromised mice (Mossallam et al., 2014). This bacteriocin-gold nanoconjugate has displayed 89.7% reduction in the number of infected intestinal cells and 93.65% reduction in the number of fecal spores, in comparison with 73.5 and 81.29%, respectively, for the free bacteriocin (Mossallam et al., 2014). In addition, the activity of incorporated bacteriocin has been sustained (with 94.26% efficiency) up to 1 week after the end of the treatment (Mossallam et al., 2014). Furthermore, this nanoconjugated bacteriocin has been shown to be safe and non-toxic, as demonstrated through behavior examinations, biochemical analysis, and histopathological screening tests (Mossallam et al., 2014).

#### Conjugation with silver nanoparticles

Silver nanoparticles are used in several applications, ranging from coating medical devices, wound dressing, coating textile fabrics, to water treatment and filtration (Furno et al., 2004; Rujitanaroj et al., 2008; Zhang et al., 2009; Dankovich and Gray, 2011). This is attributed to the broad-spectrum antimicrobial activity possessed by these nanoparticles against most clinically relevant organisms, including drug-resistant pathogens (Lara et al., 2010; Zinjarde, 2012). However, maximizing the antimicrobial efficacy of silver nanoparticles could be achieved by conjugating them to antimicrobial agents, such as bacteriocins. This approach has been demonstrated in a study conducted by Sharma et al. (2012), in which enterocin-capped silver nanoparticles (En-SNPs) have exhibited excellent efficiency against a wide range of Gram-positive and Gram-negative pathogenic bacteria. The highest level of activity of this En-SNPs has been shown against three of the most common food poisoning organisms, namely *E. coli, L. monocytogenes*, and *S. aureus* (Sharma et al., 2012). The MIC values of this En-SNPs have been shown to be 2- to 16-fold lower than that of citrate-capped silver nanoparticles (C-SNPs) (Sharma et al., 2012). Furthermore, different concentrations of En-SNPs have shown virtually no hemolytic effects against human RBCs (Sharma et al., 2012). Such results strongly motivate researchers to investigate the antibacterial activity of other bacteriocins-silver nanoparticles conjugates. However, more toxicological studies are needed to demonstrate the safety of these conjugates (Oberdörster et al., 2005).

### Incorporation into polymeric nanofibers

Nanofibers are extremely fine threads that are formed by spinning a polymer solution using a high potential electric field (Anton, 1934). Given their large surface area, small pore size, high physical stability, and powerful encapsulation ability (Doshi and Reneker, 1993; Sharma et al., 2014), nanofibers have attracted much attention as carriers for the target-specific delivery and sustained release of a variety of drugs (Luong-Van et al., 2006; Maretschek et al., 2008). In an application of this approach, an antimicrobial nanofiber wound dressing has been generated by electrospinning nisin into equimolar amounts of poly (ethylene oxide) (PEO) and poly (D, L-lactide) (PDLLA) nanofibers (Heunis et al., 2013). Nisin released from this nanofiber dressing has been shown to: (i) maintain its antistreptococcal activity *in vitro* for at least 4 days; (ii) remain active, even after storage of the formulation at 4°C for 8 months; (iii) significantly reduce the colonization of *S. aureus* in a murine excisional skin infection model; (iv) induce an almost complete wound repair, as indicated by the formation of clear fibrotic scar in the group of mice receiving the dressing; and (iv) cause no adverse effects, as revealed by histological analysis of the treated group (Heunis et al., 2013). Another similar study was carried out Heunis et al. (2011), in which nanofibers prepared using different ratios of PEO to PDLLA were used to incorporate plantaricin 423 and bacteriocin ST4SA separately. The release studies showed that a blend of PEO-PDLLA (90:10) resulted in a rapid release of Plantaricin 423 within the first 2 h, followed by a slow and constant release phase that extended for almost 8 days (Heunis et al., 2011). This pattern of release is considered ideal for infection control, since the quickly released bacteriocins will eliminate most of the microbial viable cells within the initial hours of contact, while those slowly released over the following few days are crucial for maintaining the infection under control (Heunis et al., 2011). Similarly, plantaricin 423 and bacteriocin ST4SA released from a blend of PEO-PDLLA (50:50) have been shown to maintain their AMA against sensitive bacterial strains for at least 6 days (Heunis et al., 2011).

Another example of polymeric nanofibers is the Poly-L-lactide (PLA), which is a polymeric biodegradable material that can be used as a drug delivery system once formulated in the nanoscale (Ignatius and Claes, 1996; Perez et al., 2001; Liang et al., 2006). In a study conducted by Salmaso et al. (2004), nisin has been loaded with high capacity into PLA nanoparticles, which offered a sustained-release formulation of the peptide (throughout 1000 h, depending on the pH and the salt concentration of the buffer used). Regarding the biological activity, the nisin-loaded PLA nanoparticles have displayed a potent AMA against *L. delbrueckeii* lasting up to 45 days, while the activity of the free nisin has been shown to last for 7 days only (Salmaso et al., 2004). These results indicate the usefulness of PLA nanoparticles in providing high stability and sustained release of the incorporated bacteriocin, thereby increasing their applicability in the field of food preservation. However, it must be mentioned that the toxicity of PLA is still controversial (Athanasiou et al., 1996), which requires further research on the biocompatibility of these polymers with different cell types.

Recently, a novel strategy has been implemented to maximize the benefits of bacteriocins in the biomedical field through the exploitation of nanofibers as a delivery system. This strategy depends on the electrospinning of potent bacteriocins and other beneficial substances into nanofibers to target multidrug-resistant bacteria and nosocomial pathogens. As an example of this approach, a study conducted by Ahire et al. (Ahire and Dicks, 2015) has investigated the activity of nisin after being incorporated into nanofibers prepared from PDLLA and PEO with another natural agent, called 2,3-dihydroxybenzoic acid (DHBA). This combination has shown antibiofilm activity against MRSA (Ahire and Dicks, 2015). Biofilm formation decreased by 88% following 24 h of exposure to nanofibers containing nisin and DHBA, compared to a 63% decrease for nanofibers containing only DHBA, and a 3% decrease for nanofibers containing nisin solely (Ahire and Dicks, 2015). The ability of DHBA to chelate free iron, which is needed for biofilm formation, is the proposed mechanism that explains the anti-MRSA biofilm activity (Ahire and Dicks, 2015). In another study, co-incorporation of nisin and silver nanoparticles into nanofibers has resulted in a broad AMA against a wide range of Gram-positive and resistant Gram-negative bacteria (Ahire et al., 2015). These promising results may represent a new therapeutic alternative to conventional wound dressing materials, especially against antibiotic-resistant microorganisms.

## Conclusion

Bacteriocins are a promising substitute for the currently existing antibiotics that are becoming less effective in the face of the increasing abundance of resistant organisms. However, there are several limitations that challenge the use of bacteriocins as biopreservatives / antibacterial agents in the food and pharmaceutical industries. Nanodelivery systems, such as lipid-, carbohydrate-, metal-, and polymer-based nanoparticles represent promising approaches to maximize the use of these antimicrobial peptides. Several examples of nanoformulated bacteriocins have been shown to possess better stability and a broader spectrum of antimicrobial activity in comparison with the free ones. In conclusion, nanotechnological approaches provide an interesting option toward the formulation of these antimicrobial peptides at the industry-scale level.

## Future perspectives

Bacteriocins have proven their efficiency as antibacterial agents, which explains the currently available examples of these peptides that have been commercially approved for application in the food industry. On the other hand, exploitation of bacteriocins in the health care and pharmaceutical industries is moving forward less rapidly, which is a result of a number of limitations and challenges that have yet to be solved. One of the strategies to overcome these limitations is to apply nanotechnological approaches to enhance the applicability of bacteriocins, increase their stability, and extend their antimicrobial spectrum of activity. While the different approaches to produce these formulations include encapsulation and nanomaterials conjugation, each of these approaches has its own challenges, which need to be addressed to ensure practicality of the approach. More studies are also needed to clarify whether the use of other nanodelivery systems (such as carbon nanotubes) or the combinations with nanoparticles exhibiting AMA (such as zinc oxide) could enhance the antimicrobial properties of bacteriocins. Additionally, the nature of interactions between these peptides and nanomaterials, as well as the interactions between nanoformulations of these peptides and the targeted microorganisms need to be elucidated. Further studies are also required to assess the *in vivo* efficiency and the safety of these peptides. A better understanding of these areas will pave the way toward more clinical applications of bacteriocins in the near future.

## Author contributions

HF drafted the manuscript. AK and AE revised and approved the final manuscript.

## Funding

The authors have no other relevant affiliations or financial involvement with any organization or entity with a financial interest in or financial conflict with the subject matter or materials discussed in the manuscript. This includes employment, consultancies, honoraria, stock ownership or options, expert testimony, grants or patents received or pending, or royalties. No writing assistance was utilized in the creation of this manuscript.

### Conflict of interest statement

The authors declare that the research was conducted in the absence of any commercial or financial relationships that could be construed as a potential conflict of interest.
